# The Effect of Volunteering at a Student-Run Free Healthcare Clinic on Medical Students' Self-Efficacy, Comfortableness, Attitude, and Interest in Working with the Underserved Population and Interest in Primary Care

**DOI:** 10.7759/cureus.1051

**Published:** 2017-02-23

**Authors:** Kelvin Tran, Aleksandr Kovalskiy, Anand Desai, Amna Imran, Rahim Ismail, Caridad Hernandez

**Affiliations:** 1 College of Medicine, University of Central Florida

**Keywords:** underserved, primary care, volunteering, student-run free clinic, medical student, self-efficacy, comfortableness, attitude, interest

## Abstract

**Introduction:**

The number of primary care physicians in the United States continues to lag behind the number of uninsured people. There has been a growing demand for medical students to improve their self-efficacy, comfortableness, attitude, and interest in working with the underserved and in primary care. This study aims to discern whether volunteering at a student-run, free healthcare clinic has a positive impact on these five variables of interest or not.

**Methods:**

A 95-item survey was distributed through Qualtrics Survey Software (Qualtrics, Provo, UT, USA) to medical students from the Class of 2018 and Class of 2019 at the University of Central Florida College of Medicine. They were recruited via emails, Facebook, and in-classroom announcements. Mean responses on a Likert-like scale to different survey items were collected and compared between two study cohorts: Keeping Neighbors In Good Health Through Service (KNIGHTS) Clinic volunteers and non-volunteers.

**Results:**

Results from 128 students showed no significant differences in the means between the two cohorts (p-values were not significant). When volunteers were asked the survey item, “KNIGHTS Clinic positively influenced my attitude towards working with underserved patients,” 62% strongly agreed, 26% agreed, 10% were neutral, and 2% disagreed.

**Discussion:**

Based on the results, volunteering at KNIGHTS Clinic may not have a positive impact on the five variables of interest. However, the lack of significance may also be due to certain limitations of this study addressed elsewhere in this paper. With the majority of KNIGHTS Clinic volunteers agreeing that “KNIGHTS Clinic positively influenced […their] attitude towards working with underserved patients,” there may be a positive impact of volunteering on volunteers’ attitude towards working with the underserved.

## Introduction

The shortage of primary care physicians in the United States has received considerable attention over the years as the supply of primary care physicians continues to lag behind the demand for these physicians. According to the U.S. Department of Health and Human Services, the number of primary care physicians is projected to increase by eight percent while the total demand for primary care physicians is projected to grow by 14% from 2010 to 2020 [1]. These percentages show that the growth in the supply of primary care physicians will not be adequate to meet the demand for primary care physicians by the year 2020. This shortage may be due to the fact that only about 32% of all 800,000 physicians in the United States practice primary care, and less than about 18% of current graduating medical students are expected to practice primary care [2]. Even more, “baby boomers” make up a large sector of the healthcare workforce, so with the aging and retirement of these baby-boomer physicians, it is predicted that there will be a shortage of about 91,000 physicians by 2020 and 130,000 by 2025 [3]. These shortages will indubitably affect the supply of primary care physicians and the delivery of primary care services, especially to the high number of underserved and uninsured people, which is another problem.

According to the Congressional Budget Office, there will be approximately 30 million people who will remain uninsured even after several years after the full implementation of the Affordable Care Act [4]. Through the expansion of Medicaid eligibility, establishment of Health Insurance Marketplaces, reforms that help people maintain health coverage and make private insurance plans more affordable and accessible, the Affordable Care Act did expand health coverage to millions of previously uninsured people as of 2014 [5]. However, 48% of uninsured adults noted that they are still lacking health coverage mainly due to the high cost of health insurance even under the Affordable Care Act. Additionally, undocumented immigrants are neither eligible for Medicaid nor Health Insurance Marketplace coverages [5]. It is also worth mentioning that people of color are at higher risk of being uninsured when compared to non-Hispanic Caucasian people [5].

The high number of people who are still uninsured even under the Affordable Care Act is concerning because it has a significant effect on the health care system in the U.S., specifically in regards to medical costs. Uninsured and underserved patients commonly make inappropriate and costly visits to the emergency departments with more than 120 million visits annually [6]. Many of the medical conditions that these uninsured and underserved patients had would have been more effectively treated in primary care settings, where medical services could be provided in a more cost-efficient manner that would have much less of an unfavorable effect on the bottom line for many hospitals [6]. Before the institution of strict price controls by managed care and other health plans, hospitals used to be able to shift uncompensated care costs caused by uninsured patients to the insured patients in order to make up for the difference; however, there the margin is now too little to shift these costs due to the strict price controls [7]. Overall, the problem with the increasing financial burden on the health care system that is contributed by the increasing number of uninsured and underserved people may be best approached by addressing and finding solutions to the increasing shortage of primary care physicians.

A solution to the shortage of primary care physicians may be to better promote primary care to medical students. One way to carry out this promotion is to expose medical students to a clinical setting that involves the care for the underserved and uninsured early in their medical school education. Furthermore, this clinical exposure may also increase medical students’ self-efficacy, comfortableness, attitude, and interest in working with the underserved population. At the time of this study, only Smith, et al. has explored these topics explicitly. Still, this study only explored medical students’ involvement in the provided clinical setting through an “elective medical school course” and not by means of “volunteering.” Thus, the goal of this research study is to fill in this knowledge gap by modeling the aforementioned study but exposing medical students in a volunteer clinical setting instead of an elective medical school course.

This research study focuses on the effect of volunteering—at a student-run, free healthcare clinic—on medical students’ self-efficacy (self-perception about his or her capability as student-physicians) with respect to the care for underserved individuals, comfortableness of caring for underserved population, attitude towards the underserved population, and interests in working with the underserved and working as primary care physicians after graduation. The student-run, free healthcare clinic, which the medical students in this study were exposed to, is the KNIGHTS (Keeping Neighbors In Good Health Through Service) Clinic funded by the Diebel Legacy Fund at Central Florida Foundation. The KNIGHTS Clinic is coordinated by medical students at the University of Central Florida College of Medicine and staffed by both student and physician volunteers from different areas of study who work in interdisciplinary teams alongside community collaborators [8].

## Materials and methods

### Research study's population

This research study surveyed medical students from the Class of 2018 (second-year students) and Class of 2019 (first-year students) from the University of Central Florida College of Medicine. All the medical students who participated in this study were actively enrolled in the University of Central Florida College of Medicine’s medical school curriculum. All the participants from the Class of 2019 were grouped as non-volunteers at the KNIGHTS Clinic. The participants from the Class of 2018 were divided into two groups—a group of students who have already volunteered at the KNIGHTS Clinic before this study, and another group of students who have never volunteered at the KNIGHTS Clinic prior to the study, where the latter group was analyzed like the participants from the Class of 2019.

### Recruitment methods

All the participants in this research study were recruited via emails that were sent out through the University of Central Florida College of Medicine’s Office of Assessment, in-person classroom announcements by the University of Central Florida IRB-approved co-investigators of this research study, and Facebook posts. All the participants in this study were identified via the University of Central Florida College of Medicine’s Student Directory. Only students over the age of 18 were eligible to participate in this research study. Students who were under the age of 18 and/or not an actively enrolled student were excluded from this study.

### Participants' compensation

The participants were compensated $20.00 for their time after their completion of the survey. In order to receive compensation, the participants needed to click submit on the final page of the survey, but the participants were allowed to skip questions they felt uncomfortable answering. Compensation was available by the University of Central Florida College of Medicine’s Office of Assessment after survey closure.

### Survey administration

The participants completed the survey online that was disseminated through Qualtrics Survey Software (Qualtrics, Provo, UT, USA), a web-based system for surveys. The online survey was made available to be completed on any electronic devices with internet access at any time and location convenient for the participants. The survey was sent out by the University of Central Florida College of Medicine’s Office of Assessment. Each participant’s participation only lasted as long as it took him or her to complete the survey, which should not have been longer than 20 minutes.

### Survey content

Both volunteering and non-volunteering groups of medical students were given the same survey. The survey was a 95-item survey instrument: demographic information with 11 items; volunteer clinical experiences prior to medical school with six items; knowledge/skill/self-efficacy/comfort level/interest/attitude regarding the underserved with 22 items; interpersonal reactivity index (empathy) questions with 28 items; interdisciplinary comfort with 16 items; and KNIGHTS Clinic specific questions with 12 items. Depending on the participants’ responses about his or her clinical volunteer experience, the number of survey questions responded by the participants varied from the 95 items listed above. This 95-item survey was designed to investigate more research topics that are of interest to other co-investigators in the University of Central Florida IRB-approved protocol. Consequently, the survey also includes items that were not relevant to this particular research study. The sections of the survey that are relevant to this research study are the aforementioned sections on the demographic information, knowledge/skill/self-efficacy/comfort level/interest/attitude regarding the underserved, and KNIGHTS Clinic specific questions. The section of most interest is the section about the knowledge/skill/self-efficacy/comfort level/interest/attitude regarding the underserved. Only this section of the survey was principally adopted from the Smith, et al. study that was mentioned earlier in this paper with minimal changes. The only difference between the survey questions in this section of this research study and the survey questions in the original survey was the different populations addressed by the survey questions. The original survey in Smith, et al. study asks about self-efficacy, comfort level, interest, and attitude towards working with “the homeless” and “underserved minority families [9],” while this research study asks about the following populations: undocumented individuals, uninsured individuals, underserved women, and the underserved. This research study only analyzed 14 questions in this section of the survey about the participants’ self-perception of their self-efficacy, comfort level, interest, and attitude towards working with the specified populations and the participants’ interest in being a primary care physician after graduation. Also, one out of 12 questions from the section on the KNIGHTS Clinic specific questions was analyzed in this research study to explicitly assess the effect of volunteering at the KNIGHTS Clinic on the participant’s self-perceptions of their attitude towards the underserved. The participants’ responses to the questions from the section about knowledge/skill/self-efficacy/comfort level/interest/attitude regarding the specified populations were obtained using a 7-point Likert-Type Scale, from one “Not at all” to seven “A great deal.” The participants’ responses to the questions in the section on the KNIGHTS Clinic specific questions were obtained using a 6-point Likert-Type Scale, from one “Strongly Disagree” to six “Not Applicable.”

### Data management

The data for this research study were not transported but were shared with the co-investigators in the University of Central Florida IRB-approved protocol for this research study via Excel File. For quality control, all files were password protected. The data included participants’ responses to the survey measures. The University of Central Florida College of Medicine’s Office of Assessment was responsible for receipt and transmission of the de-identified data to the research team. The data were shared via Qualtrics as much as possible to allow for storage of the data within the platform. The data for this research study were linked with the participants’ names and email addresses and were de-identified prior to analysis and before given to the research team. Identifiable data will be stored by the University of Central Florida College of Medicine’s Office of Assessment in password-protected computers, and all data will be stored according to the University of Central Florida’s regulations for five years.

### Data analysis

Descriptive statistics were used to report the demographics of the participants in this research study and are represented in frequency and percentage. The effect of volunteering at KNIGHTS Clinic on the participants regarding the five variables of interest in this research study was assessed by comparing the mean scores of the participants’ responses across two groups: “Medical students with volunteering experience at KNIGHTS Clinic” versus “Medical students without volunteering experience at KNIGHTS Clinic.” Continuous data (survey questions) are represented in mean and standard deviation. Independent sample T-test was used for analysis. Statistical significance was tested using a p-value of less than 0.05. Statistical analyses were conducted using SPSS 23.0 (IBM; Armonk, NY, USA).

## Results

### Study population

A total of 136 medical students were surveyed in this research study. However, data from eight students were intentionally excluded from analysis because these participants did not answer enough survey questions relevant to this research study. Consequently, the final number of students, whose data were actually used in the analysis, is 128, with n = 73 from the Class of 2018 reported as “M2” and n = 55 from the Class of 2019 reported as “M1.” The majority of participants were second-year medical students (M2), accounting for 57% of the participation. Most of the participants were identified as female (53.1%) and “White” (56.3%). Additionally, the majority of the participants were in the 20-25 age group (74.3%). Two students did not provide information about their ages in their survey responses (reported as “Missing”). These demographic characteristics are reflected in Table 1.

**Table 1:** Demographic Characteristics of Study Population M1: First-year medical students; M2: Second-year medical students

Characteristic	Frequency	Percent
Year in Medical School		
M1	55	43.0
M2	73	57.0
Total	128	100.0
Gender		
Male	59	46.1
Female	68	53.1
Other	1	0.8
Total	128	100.0
Racial Ethnic Group		
African American	0	0
Asian/Pacific Islander	40	31.3
Hispanic	7	5.5
White	72	56.3
Other	9	7.0
Total	128	100.0
Age		
20-25	95	74.3
26-30	25	19.5
31-35	4	3.2
36-40	2	1.6
Missing	2	1.6
Total	128	100.0

The majority of the participants in this study were not active volunteers at the KNIGHTS Clinic, accounting for 60.9% of the participation. The participants in the “KNIGHTS Volunteer” study group accounted for 39.1% of the participation in this research study. Consequently, the non-volunteer study group had 56% more participants relative to the KNIGHTS Clinic volunteer study group. These volunteering percentages are reflected in Table 2.

**Table 2:** Study Population’s Volunteer Status KNIGHTS: Keeping Neighbors In Good Health Through Service

Group	Frequency	Percent
Not a KNIGHTS Volunteer	78	60.9
KNIGHTS Volunteer	50	39.1
Total	128	100.0

### Participants' responses

No statistical significance in the difference between the means of the responses to the survey questions by the KNIGHTS Clinic volunteers and the mean values from the non-volunteers could be established (p-values were not significant). The survey questions with the corresponding mean values, where the means from the KNIGHTS volunteer group are greater than the means from the non-volunteer group, are bolded in Table 3 below: survey questions 1, 3, 5, 7, 13, and 14 displayed in the table have mean differences of 0.03, 0.1, 0.27, 0.4, 0.1, and 0.45, respectively, in the mean values compared between the two study groups. The greatest mean differences along with the lowest p-values compared to the responses from the other survey questions were observed for survey questions 7 and 14, which address the following questions, respectively: “I feel capable of caring for underserved population” and “My interest in being a primary care physician is.” All other non-bolded survey questions have the means from the non-volunteer study group being greater than the means from the KNIGHTS volunteer study group. Moreover, with the exception of survey questions 4, 9, and 10, the results from the rest of the survey questions in the table show that the standard deviations from the non-volunteer study group are higher than the standard deviations from the KNIGHTS volunteer study group. These results to the survey questions are reflected in Table 3.

**Table 3:** Results from Participants’ Responses to Survey Questions The bolded survey questions with their associated values have significances addressed elsewhere in the paper. KNIGHTS: Keeping Neighbors In Good Health Through Service; Std.: Standard

Survey Question	KNIGHTS Volunteer (Mean)	Non-Volunteer (Mean)	KNIGHTS Volunteer (Std. Deviation)	Non-Volunteer (Std. Deviation)	p-value
I feel capable of caring for undocumented individuals (1)	4.18	4.15	1.494	1.894	0.934
I feel comfortable caring for undocumented individuals (2)	4.76	4.92	1.768	1.800	0.615
I feel capable of caring for underserved women (3)	4.40	4.30	1.641	1.702	0.740
I feel comfortable caring for underserved women (4)	5.02	5.17	1.684	1.673	0.626
I feel capable of caring for the uninsured population (5)	4.76	4.49	1.575	1.885	0.389
I feel comfortable caring for the uninsured population (6)	5.10	5.36	1.686	1.698	0.407
I feel capable of caring for underserved population (7)	4.82	4.42	1.521	1.931	0.198
I feel comfortable caring for underserved population (8)	5.18	5.31	1.521	1.931	0.659
My attitude towards the care of undocumented individuals is (9)	5.50	5.67	1.502	1.213	0.491
My attitude towards the care of uninsured population is (10)	5.82	5.94	1.304	1.109	0.591
My attitude towards the care of underserved women’s health is (11)	6.02	6.04	1.010	1.050	0.924
My attitude towards the care of underserved population is (12)	6.04	6.05	1.029	1.043	0.952
My interest in working with the underserved after I graduate is (13)	5.22	5.12	1.327	1.414	0.666
My interest in being a primary care physician is (14)	3.82	3.37	1.535	1.676	0.130

**Figure 1:**
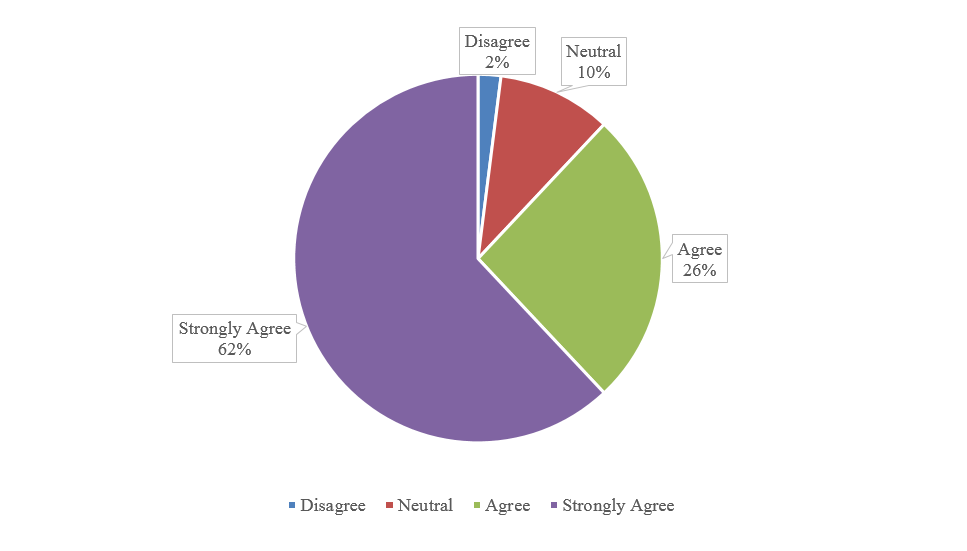
KNIGHTS volunteers’ responses to the question, “The KNIGHTS Clinic positively influenced my attitude towards working with underserved patients" The color-coded shaded areas of the graph represent the different responses from KNIGHTS volunteers to a specific survey question. KNIGHTS: Keeping Neighbors In Good Health Through Service.

When KNIGHTS volunteers were asked whether the “KNIGHTS Clinic positively influenced […their] attitude towards working with underserved patients” or not, the majority of the volunteers agreed with 62% strongly agreed and 26% agreed with this survey question. A total of 10% of the volunteers were neutral and two percent disagreed with the survey question. These responses from the KNIGHTS volunteers are reflected in Figure 1.

## Discussion

The null hypothesis of this research study is as follows: volunteering at a student-run, free healthcare clinic has no effect on medical students' self-efficacy, comfortableness, attitude, and interest in working with the underserved population and interest in primary care compared to non-volunteering. As mentioned in the “Results” section, no statistical significance in the difference between the means of the responses to the survey questions by the KNIGHTS Clinic volunteers and the mean values from the non-volunteers could be established; thus, this null hypothesis could not be rejected. Consequently, based on the results from this research study, there may be no effect of volunteering at a student-run, free healthcare clinic on medical students' self-efficacy, comfortableness, attitude, and interest in working with the underserved population and interest in primary care.

Nonetheless, despite the careful design of this research study, there are a few limitations to this study that could have contributed to the lack of statistically significant results that need to be addressed from hindsight. First of all, of the total number of analyzed participants in this research study, only 39.1% were KNIGHTS volunteers while 60.9% were non-volunteers. This large difference in the sample size with the non-volunteer study group having 56% more participants relative to the KNIGHTS Clinic volunteer study group may have permitted the non-volunteer study group to end up with higher mean values in the responses to the comparative survey questions. Secondly, as mentioned in the “Results” section, the standard deviations in the responses from the majority of the survey questions from the non-volunteer study group were higher than the standard deviation values observed from the KNIGHTS volunteer study group. This suggests that these responses from the non-volunteer group were more spread out over a wider range of values on the 7-point Likert Scale for these survey questions compared to the KNIGHTS volunteer study group.

Moreover, the number of exposures in term of volunteering hours for each KNIGHTS volunteer could have been useful in this research study. The effects of volunteering at KNIGHTS Clinic may not have been significant if the KNIGHTS volunteer did not volunteer often or consistently. Additionally, the roles of each KNIGHTS volunteer at the clinic were unknown. Some volunteers like those who were assigned as student pairs to see patients may have been able to interact with patients much more than other volunteers like those who were assigned to manage the front desk or the lab station; thus, the effects of volunteering at KNIGHTS Clinic may also have been different for each volunteer based on his or her duties carried out while volunteering. These variations in clinical exposures (number of volunteer sessions and hours) and clinical duties within the KNIGHTS volunteer study group may have also impacted the responses to the survey questions from this study group alone. The KNIGHTS volunteers who had low clinical exposure and/or low patient interactions may have lowered the mean values.

These limitations should be addressed and changes should be made in future studies. First of all, the sample size of the two study groups should be made fairly comparable in order to avoid the effect from a sample size difference. Secondly, the KNIGHTS volunteer study group should be further analyzed based on clinical exposure by having data on each volunteer’s total volunteering hours in order to avoid the effect of a variation in responses by clinically low-exposed volunteers compared to clinically high-exposed volunteers. Thirdly, KNIGHTS volunteer study group should also be further analyzed based on patient exposure by having data on each volunteer’s clinical role at each volunteer session in order to avoid the effect of a variation in responses by volunteers with low patient interaction compared to those with high patient interaction. Lastly, the additions of the questions, “The KNIGHTS Clinic positively influenced my self-efficacy of working with underserved patients,” “The KNIGHTS Clinic positively influenced my comfortableness towards underserved patients,” and “The KNIGHTS Clinic positively influenced my interest in working with underserved patients” should be made to the “KNIGHTS Clinic Specific Questions” section of the survey in order to further expand on the positive results from the responses to the question, “The KNIGHTS Clinic positively influenced my attitude towards working with underserved patients,” that was explored in this research study.

Even though no statistically significant effect from volunteering at the KNIGHTS Clinic on the five variables of interest in this study could be concluded from the results of this research study as reflected in Table 3, the volunteering experience may still have a positive effect on the KNIGHTS volunteers. As mentioned in the “Results” section, when the KNIGHTS volunteers were asked whether the “KNIGHTS Clinic positively influenced […their] attitude towards working with underserved patients” or not, the majority of them either strongly agreed or agreed with this survey question. Therefore, there may actually be a positive effect of volunteering at KNIGHTS Clinic on the volunteers’ attitude towards working with the underserved as concluded by the aforementioned results.

## Conclusions

Although the results of this research study did not provide evidence for improvement in medical students' self-efficacy, comfortableness, attitude, and interest in working with the underserved population and interest in primary care through volunteering at the KNIGHTS Clinic, the importance of the research question explored in this research study should not be undermined. This research study has yielded useful data for future studies since there is a lack of research studies and, hence, a limited knowledge about this research topic. This research study could be used as a starting point to fill in this knowledge gap about the effect of “actual volunteering” specifically at KNIGHTS Clinic on medical students’ self-efficacy with respect to the care for underserved individuals, comfortableness in caring for underserved population, attitude towards the underserved population, and interests in working with the underserved and working as primary care physicians. Further knowledge about this research topic is important because it would help to decide if it would be beneficial or not to incorporate volunteering in a student-run, free healthcare clinic into a medical school’s curriculum in hope of preserving medical students’ positive attitude towards the underserved and better promote primary care to help reduce the primary care shortage.
